# Pregnancy and Childbirth in the COVID-19 Era—The Course of Disease and Maternal–Fetal Transmission

**DOI:** 10.3390/jcm9113749

**Published:** 2020-11-21

**Authors:** Agnieszka Irena Mazur-Bialy, Daria Kołomańska-Bogucka, Sabina Tim, Marcin Opławski

**Affiliations:** 1 Department of Biomechanics and Kinesiology, Faculty of Health Science, Jagiellonian University Medical College, Grzegorzecka 20, 31-531 Krakow, Poland; daria.kolomanska@doctoral.uj.edu.pl (D.K.-B.); sabina.tim@doctoral.uj.edu.pl (S.T.); 2 Department of Gynecology and Obstetrics with Gynecologic Oncology, Ludwik Rydygier Memorial Specialized Hospital, Zlotej Jesieni 1, 31-826 Kraków, Poland; oplawski.m@gmail.com

**Keywords:** COVID-19, SARS-CoV-2, pregnant women, maternal–fetal transmission, vertical transmission, fetus, labor

## Abstract

From the beginning of the Coronavirus Disease 2019 (COVID-19) pandemic, special attention has been paid to pregnant women and to monitoring comorbidities, such as gestational diabetes and hypertension, which could increase their risk of disease and death. The purpose of this review is to synthesize the available knowledge on the course of COVID-19 in pregnant women as well as the risk of maternal–fetal transmission. The study indicated that the course of COVID-19 is worse in pregnant women who are more often admitted to intensive care units or who require mechanical ventilation than nonpregnant women with COVID-19. Some symptoms, such as dyspnea and cough, were similar to those observed in nonpregnant women, but fever, headache, muscle aches, chills, and diarrhea were less frequent. A study revealed that premature delivery and cesarean section were more common in pregnant women diagnosed with COVID-19. In addition, recent studies confirm the possibility of intrauterine maternal–fetal transmission by positive genetic tests and the presence of IgM in newborns just after delivery; at the moment, the probability of transmission through mother’s milk is inconclusive. Considering all the above, a severe acute respiratory syndrome coronavirus-2 (SARS-CoV-2) infection is an important factor that threatens the health and life of both the mother and the fetus, but further studies are still needed.

## 1. Introduction

At the end of 2019, a lot of people in Wuhan, China, suffered from pneumonia of an unknown cause [1] which, in February 2020, was named as Coronavirus Disease 2019 (COVID-19). It is caused by severe acute respiratory syndrome coronavirus-2 (SARS-CoV-2) and manifests as acute respiratory failure [2]. On 11 March 2020, the World Health Organization (WHO) declared that COVID-19 is a pandemic [3]. As of 6 November 2020, COVID-19 was diagnosed in 48,786,440 people from 219 countries or territories, while the number of deaths due to the disease was 1,234,839 [4] and continues to grow.

The risk of SARS-CoV-2 infection and development of COVID-19 symptoms increase with age and the prevalence of comorbidities [5]. Previous studies suggested that pregnant women are not more susceptible to SARS-CoV-2 infection than the general population [6]. However, the presence of comorbidities (e.g., diabetes and chronic hypertension), which often appear in the second trimester of pregnancy [7,8], as well as increased maternal age and high BMI may increase their risk of developing severe COVID-19 symptoms [8]. A report published on 26 June 2020 mentioning that 8207 pregnant women were confirmed to have SARS-CoV-2 infection altered this perception as it revealed that pregnant women may have an increased risk of developing severe symptoms of COVID-19 as compared to nonpregnant patients [9,10]. These observations are also confirmed by a meta-analysis by Allotey et al. [8] and a large meta-analysis by Khali et al. [11]. SARS-CoV-2 infection was reported in 9% [9] to 13.5% of women giving birth [12], with the asymptomatic course of the disease in pregnant women estimated up to 90% [12]. Moreover, because of severe COVID-19 symptoms, pregnant women were significantly more often admitted to the intensive care unit and required mechanical ventilation than nonpregnant women [9] and healthy pregnant women [11]. Nevertheless, the risk of death was similar in both groups and ranged in various studies from 0.1% [8] per 0.8% [13] to 0.9% [11]. The abovementioned outcomes were more frequently observed in pregnant women aged 35–44 years than in those aged 15–34 years [9] and with comorbidities [11]. Scientists also point out that pregnant women with symptomatic COVID-19 are more likely to experience spontaneous premature births than healthy women and that their newborn babies more often require support in neonatal departments [8]. In addition, studies also show a greater risk of select races to SARS-CoV-2 infection. Analysis of the data to date suggests that pregnant women who are Hispanic [14,15], Black [15], or Latino [14] may be at a higher risk of SARS-CoV-2 infection.

The purpose of this narrative review is to present the available knowledge on the course of COVID-19 in pregnant women as well as on the risk of maternal–fetal transmission, the management of an infected pregnant woman, breastfeeding, and the emotional state of pregnant women. For this purpose, PubMed, Embase, Web of Science, and Google Scholar databases were searched using appropriate keywords. Literature review was performed for relevant studies published until 22 October 2020.

### 1.1. Immunological Changes during Pregnancy and COVID-19 Infection

Pregnancy is a specific immunological state that requires the development of tolerance to the allogeneic fetus while maintaining the ability to protect against pathogenic infection [16], which may increase the susceptibility of pregnant women to infections [17,18]. The period of pregnancy is associated with significant changes in the areas of innate, cellular, and adaptive immune responses. These changes can be divided into three states: an initial proinflammatory state that allows embryo implantation, an anti-inflammatory state that allows fetal growth and inhibits the induction of labor, and a second proinflammatory state in the third trimester that aids in delivery [16]. From the first trimester, the levels of blood monocytes, granulocytes, and dendritic cells (DCs) increase, with a peak occurring in the second trimester. Starting from the 13th week of pregnancy, blood monocytes undergo a functional change, because of which the secretion of IL-1β and IL-12 increases and the potential for TNF-α secretion decreases. Moreover, a state of moderate lymphopenia is also observed due to a decrease in the number of CD4 and CD8 T lymphocytes. However, the level of Treg lymphocytes is increased. Moreover, the level of natural killer (NK) cells, which play an important role in angiogenesis and vessel formation in the first trimester and which subsequently constitute nearly 70% of deciduous leukocytes in early pregnancy, decrease in the circulating blood. In the third trimester, the number of B lymphocytes also decreases [18]. These changes create an increased sensitivity to develop infections, especially in the first trimester, but at any point in pregnancy, infections by virus can negatively affect pregnant women [19]. Beyond disease symptoms, these infections increase the risk of developing complications in the mother and newborn (e.g., premature delivery, restriction of intrauterine growth, and spontaneous abortion) [20]. Previous study indicated that many viruses cause more severe symptoms when they develop in pregnant women, including e.g., hepatitis E virus (HEV) [21], dengue virus [22], and H1N1 influenza virus [23]. Compared to nonpregnant adult women, pregnant women are more likely to develop infections caused by respiratory pathogens and they are more likely to have a severe course of the disease due to immunosuppressive state and physiological changes in pregnancy, higher diaphragm position, increased oxygen demand, and edema of the respiratory tract mucosa, which makes them more vulnerable to hypoxia [10].

Worldwide observations show that the incubation time of the SARS-CoV-2 virus ranges from 2 to 14 days post infection, and clinically, patients can be asymptomatic or can develop moderate to severe symptoms [24]. The ACE2 (angiotensin-converting enzyme 2) receptor plays an important role in the infection of target cells by SARS-CoV-2 [25]. Expression of this receptor is noted in type 2 alveolar cells as well as in the kidneys, esophagus, and heart cells [26] and in small amounts in monocytes and macrophages [27]. During mild SARS-CoV-2 infection, both innate and adaptive immune responses cooperate with each other synergistically; however, in the case of a severe course of infection, marked dysregulation in immune responses is noted [28]. In severe vs. nonsevere COVID-19 patients, significant increases in total leukocyte count, especially neutrophils, and in the neutrophil-to-lymphocyte ratio (NLR) were observed with a simultaneous decrease in the levels of macrophages, basophils, and eosinophils. In addition, most patients in severe condition developed marked lymphopenia with significant reduction in lymphocytes, mainly CD4+ T cells, CD+8 T cells, regulatory T cells, NK cells, and B cells. In mild cases of COVID-19, this dysregulation is not as pronounced as that observed in severe cases [28,29]. Because of this imbalance, there is a massive release of proinflammatory cytokines, a phenomenon termed as a “cytokine storm,” while mechanisms of regulation and silencing of the immune response seem to be impaired. Studies show a particularly high increase in IL-6 level, which is significantly higher in acute, fatal cases than in milder cases. COVID-19 may alter immune responses at the maternal–fetal interface and thereby affect the well-being of mothers and infants [16]. Studies also indicate a disturbance and decrease in the Treg/Th17 ratio caused by a significant increase in the Th17 population in the acute course of COVID-19. This aspect is particularly important for pregnant women, in whom Treg cells allow the development of an allogeneic fetus, while Th17 cells protect against infection by pathogens. Proper Treg/Th17 ratios are crucial for implantation of the embryo and healthy pregnancy; therefore, this balance is shifted toward Treg cells to ensure maternal–fetal immune tolerance [30,31]. Therefore, a reduction in the Treg/Th17 ratio is associated with pregnancy complications such as miscarriage, preeclampsia (PE), and preterm labor [31]. Keeping this in mind, the reduction in the number of Treg cells observed during COVID-19 along with an increase in the number of Th17 cells may at least in part, particularly in more severe cases, be the cause of potential miscarriages, premature births, or PE.

### 1.2. COVID-19 Diagnosis in Pregnant Women

Pregnant women, like any other person, should be examined for fever and respiratory infections before delivery. Ideally, screening procedures should be performed before admitting a patient in the maternity ward [32]. It is necessary to confirm the diagnosis of SARS-CoV-2 infection by performing a real-time reverse transcription polymerase chain reaction (RT-PCR) assay [33]. According to WHO recommendations, SARS-CoV-2 virus detection can be performed using nasopharyngeal and oropharyngeal swabs in ambulatory patients, from sputum, and/or from endotracheal aspirate or bronchoalveolar lavage in patients with severe forms of the disease [34]. According to a meta-analysis by Böger et al. [35], RT-PCR tests performed with rectal stools/swab, urine, and plasma were less sensitive in detecting the virus than tests performed with sputum (97.2%, 95% CI 90.3–99.7%), saliva (62.3%, 95% CI 54.5–69.6%), nasopharyngeal aspirate/swab, and throat swab (73.3%, 95% CI 68.1–78.0%). The difference in sensitivity between different types of smears may depend on the degree of disease progression [36]. Nevertheless, some studies indicate that the risk of obtaining false-negative results for COVID-19 is approximately 30–40% [37]. If the nucleic acid of SARS-CoV-2 is not detected in two consecutive tests performed at least 24 h apart, the result can be considered negative [17].

COVID-19 diagnostics include also X-rays and computed tomography (CT) of the chest. However, a review by Salameh et al. noted that, on the basis of CT examination, a positive result of SARS-CoV-2 infection can be obtained in 86% (95% CI: 72–94) of COVID-19 patients and in 82% (95% CI: 44–96) of healthy people. The sensitivities of chest CT and X-rays in patients with confirmed SARS-CoV-2 infection were 93.1% (95% CI: 90.2–95.0) and 82.1% (95% CI: 62.5–92.7), respectively. On the other hand, in the studies of suspected COVID-19 cases, the sensitivity and specificity of CT were 86.2% (95% CI: 71.9–93.8) and 18.1% (95% CI: 3.71–55.8), respectively. Therefore, CT examination cannot clearly differentiate SARS-CoV-2 infection from other respiratory diseases [38]. Lung imaging using ultrasound can also be performed in pregnant women [37]. However, the results of each imaging examination (CT, X-rays, and ultrasonography (USG)) should be carefully interpreted [38].

Due to high risk of developing asymptomatic infection, the WHO recommends that all pregnant women who are in contact with a person with SARS-CoV-2 should be monitored [39]. Bianco et al. [40] screened pregnant women and the persons who were to accompany them during delivery for the development of COVID-19 1 day before the scheduled delivery date. They reported that asymptomatic COVID-19 was diagnosed in 15.5% of pregnant women and in 9.6% of accompanying people [40].

### 1.3. Symptoms of COVID-19 among Pregnant Women

Numerous reports indicated that the main symptoms of COVID-19 in pregnant women are similar to those observed in nonpregnant women with COVID-19 and in the general population [6,8,10]; however, some of them occur less frequently during pregnancy [9]. In both groups of women, the incidence of cough and shortness of breath was comparable (51.8% vs. 53.7% and 30.1% vs. 30.3%, respectively, in pregnant and nonpregnant women). On the other hand, fever (34.3% vs. 42.1%), headache (40.6% vs. 52.2%), muscle aches (38.1% vs. 47.2%), chills (28.5% vs. 35.6%), and diarrhea (14.3% vs. 23.1%) were much less frequent in pregnant than nonpregnant women [9]. Also, studies by Allotey et al. [8] emphasize that fever and myalgia are less often observed in pregnant women with COVID-19. On the other hand, according to a review by Teles Abrao Trad et al. [19], the most common symptoms of SARS-CoV-2 infection in pregnant women are fever (57.6%), cough (31.7%), dyspnea (13.7%), and gastrointestinal disorders (20.9%). In turn, in the research by Brandt et al. [15], it was shown that, in pregnant women with a mild course of COVID-19, the most common symptoms were fever (24.1%), cough (25.9%), and myalgia (9.3%), while in pregnant women with severe or critical COVID-19, the most common symptoms were cough (100%), shortness of breath (85.7%), and fever (71.4%). Compared to the control group (no SARS-CoV-2 infection), women with severe or critical COVID-19 had a higher rate of comorbidities (42.9% vs. 24.6%) [15]. The symptoms of COVID-19 also include fatigue; headache; and throat, limb, or joint pain [41]. In addition, in the perinatal period and immediately after it, intensification of the disease symptoms is often observed. Khoury et al. [42] showed that 61.4% of pregnant women did not show any symptoms of COVID-19 at the time of admission to the hospital. However, during childbirth, 26.5% of them developed mild symptoms, 26.1% developed severe symptoms, and 5% developed critical symptoms. Of those women with severe and critical symptoms, 52.4% and 91.7%, respectively, underwent cesarean section [42]. Acute worsening of symptoms or their occurrence in the perinatal or postpartum period in asymptomatic or mildly symptomatic women is frequently reported in the literature [43,44,45]. Research has also shown that symptomatic pregnant women had a higher chance of experiencing preterm delivery [46,47] or undergoing cesarean section than asymptomatic women [46]. The frequency of spontaneous preterm births and overall preterm births in women with COVID-19 is higher than in healthy ones; has risen to 6 and 17%, respectively [8]; and, according to Hanna et al. [14], may be associated with a storm of pro-inflammatory cytokines accompanying the acute course of SARS-CoV-2 infection. Moreover, multinational studies emphasize that the perioperative SARS-CoV-2 infection significantly increased the risk of postoperative pulmonary complications, which were observed in half of the patients, and also significantly increased their mortality. Postoperative pulmonary complications after obstetrics surgery occurred in 49% of patients with SARS-CoV-2 infection [48]. For this reason, the cesarean section should be carefully discerned and used in justified cases, more so as there is no direct evidence of an increased risk of viral transmission upon vaginal delivery. It should be also emphasized that the incidence of adverse outcomes in pregnant women increases with the severity of COVID-19 symptoms [15]. Research by Brandt et al. noted that, in pregnant women with mild COVID-19 symptoms, similar obstetric results to healthy ones were obtained. Nevertheless, pregnant women with severe/critical COVID-19 had more adverse obstetrical outcomes—e.g., earlier gestational age of delivery; a higher risk of preterm labor; and a higher risk of antenatal admissions, cesarean sections, chorioamnionitis, preeclampsia, and persistent category 2 fetal heart rate tracing despite intrauterine resuscitation [15].

It should be also noted that as many as 42.9% of newborns of infected mothers suffered from respiratory distress compared to 10.9% in the group of children born to healthy mothers. Poor oral intake had 35.7% and 6.3% of children born of SARS-CoV-2-positive and -negative mothers, respectively. Newborns born to mothers with severe/critical COVID-19 have a lower birthweight compared to newborns born to women with mild disease or healthy ones. They can also get a lower score on the 1-min and 5-min APGAR scales (Appearance, Pulse, Grimace, Activity, Respiration) [15]. Moreover, only 20% of mothers with COVID-19 had skin-to-skin contact with their babies compared to 76.6% in the group of healthy mothers. Most infants born to infected mothers were isolated (73.3%) compared to those born to healthy women (23.4%) [49].

Attention should also be paid to cases of miscarriage and perinatal death observed in women positive for SARS-CoV-2. In a secondary analysis [50], a multinational study [13] conducted from 1 February 2020 to 30 April 2020 on a population of 22 high-income countries among 388 pregnant women with an RT-PCR-confirmed SARS-CoV-2 infection (nasal and pharyngeal swab) highlights a significant increase in the risk of complications and the incidence of composite-adverse fetal outcome if mothers underwent infection in the first trimester (35.3 vs. 2%, *p* < 0.001), with spontaneous first-trimester abortion reaching the level of 19.4% (95% CI: 9.2–36.3). However, the overall incidence of perinatal death was 4.2% (95% CI: 2.3–7.3) with 2% of neonatal death (95% CI: 0.9–4.6). Cases of miscarriage and perinatal death in asymptomatic and symptomatic women are described in the literature, and despite the fact that their frequency is not high, they should be considered as one of the potential negative outcomes. Richtmann et al. [51] showed cases of fetuses dying at 21–38 weeks of pregnancy. In one case, SARS-CoV-2 was detected in the amniotic fluid, and in two cases, it was detected in placental tissues. One of the fetuses was diagnosed with neutrophils in the alveolar spaces, indicating COVID-19 infection. All five examined women had intense placental inflammation, intense infiltration of neutrophils and lymphocytes, and mixed intervillositis/villitis. There were no other significant clinical or obstetric abnormalities found which were capable of inducing miscarriage [51]. In July, Hachem et al. [52] reported a case of early miscarriage in the 20th week of pregnancy, which was described as “an inaugural manifestation of COVID-19”. The patient came to the gynecological unit with vaginal bleeding and uterine contraction, without fever or COVID-19 symptoms. Laboratory tests showed lymphocytopenia (0.87 Giga/L), and high C-reactive protein (137 mg/L) and ferritin (261 mg/L) levels, and the genetic test for SARS-CoV-2 was positive in the mother but not in the placenta. Immediately after delivery, the mother developed severe COVID-19 respiratory symptoms, pneumonia in an angio-TC invaded nearly 50% of the lungs. Critical COVID-19 can cause multiorgan damage in the mother, as a result of which the newborn may die shortly after its birth, as described by Li et al. [43], or in case of infection require rapid respiratory support [44]. Table 1 shows the symptoms of COVID-19 among pregnant women reported by different studies.

### 1.4. Intrauterine and Transplacentar Transmission

According to the official WHO data, the incubation period for COVID-19 (the time between exposure to the virus and the appearance of symptoms) is on average 5–6 days, but it can last even up to 14 days [60]. Transmission of SARS-CoV-2 in humans occurs primarily via droplets released while talking, coughing, or sneezing [61]. The risk of viral transmission is higher in the case of short contact with a symptomatic person than with an asymptomatic person [62]. SARS-CoV-2 is also transmitted when a person touches the surfaces contaminated with the virus [63]. Therefore, the COVID-19 prevention guidelines for pregnant women include avoiding contact with sick people, crowded places, public transport, and unnecessary travel. It is also important that pregnant women constantly maintain proper personal and social hygiene [16].

Current studies indicate that the probability of vertical SARS-CoV-2 transmission from mother to newborn ranges from 3% [64] to 8% [65]. A study by Wang et al. [66] showed that, after 36 h of birth, a positive result was obtained for a neonate from a throat swab in the RT-PCR test. COVID-19 was also diagnosed in the mother. However, SARS-CoV-2 was not detected in the samples of umbilical cord blood, placenta, and breast milk. In this case, despite the fact that no viral genes were detected in the umbilical cord blood as well as in the placental tissues, intrauterine transmission of SARS-CoV-2 cannot be excluded. Thus, if the viral load is insufficiently high, a false-negative result can be obtained [66]. In turn, Dhawan et al. [67] reported a case of infection of a newborn with SARS-CoV-2 from a mother in whom the infection progressed asymptomatically (including no upper respiratory tract infection, fever, or rash). The baby was born prematurely by caesarean section. Immediately after delivery, the newborn developed respiratory distress, and the COVID-19 infection was confirmed at 23 h of life (nasopharyngeal swab RT-PCR) [67]. However, symptoms of SARS-CoV-2 infection in a newborn do not have to appear immediately after birth but in the following days [68]. Vivanti et al. [69] demonstrated the transmission of SARS-CoV-2 across the placenta from an infected mother to her child (viral load was significantly higher in the placental tissue than in the amniotic fluid or the blood of both the mother and newborn). Nasopharyngeal and rectal swabs were collected in the first hour of the child’s life and, again, on the 3rd and 18th days, and RT-PCR test conducted each time showed a positive SARS-CoV-2 result. Moreover, the viral loads indicated by the RT-PCR curves generated for the neonatal nasopharynx swabs taken at 3 and 18 days of age were higher than on day 1 of the study [69]. In turn, in the studies of Kulkarani et al. [70], the presence of SARS-CoV-2 was demonstrated in the swab of the nasopharynx, cord stump, and the placenta at 12 h of a newborn’s life. The mother’s RT-PCR test was performed on the 2nd and 5th days postpartum, and in both cases, negative results were obtained. Severe symptoms of infection in the child (e.g., fever, icterus, and poor feeding) appeared in 38 h of life. On the other hand, the mother had a serological test on the 2nd day of the postpartum period (negative result). The test was repeated on the 10th and 21st days after delivery, and then, the test was positive for the presence of antibodies. In a newborn, a similar result was obtained at 21 days of age [70].

Patanè et al. [71] diagnosed COVID-19 in two newborns. In one of them, positive results indicating the presence of SARS-CoV-2 were obtained within 24 h of birth, while in the other, positive results were obtained only on day 7 (the child was isolated from the infected mother throughout this period). In both cases, the maternal placenta showed chronic intervillositis, with the presence of macrophages [57]. Penfield et al. [72] demonstrated the presence of SARS-CoV-2 genes in the membrane and placental samples in three newborns. However, the nasopharyngeal swabs, which were examined at least twice in each newborn, were not tested positive for the SARS-CoV-2 infection [72]. In turn, Ferraiolo et al. [73] presented a case of asymptomatic infected pregnant women with SARS-CoV-2. At the time of cesarean section, the results of the COVID-19 tests were unknown. Consequently, the baby was separated from the mother after birth. After delivery, the mother obtained positive results for the presence of SARS-CoV-2 in the nasopharyngeal swab and anti-SARS-CoV-2 IgG. In newborns, nasopharyngeal swabs collected immediately after birth obtained inconclusive results; the next two tests were negative, while the placental swabs were positive for SARS-CoV-2 RNA [73]. In the case of an acute course of COVID-19, lymphohistiocytic villitis may develop, possibly caused by infection with SARS-CoV-2 of the placenta. The symptomatic form of COVID-19 is characterized by the most pronounced inflammatory response as well as the presence of SARS-CoV-2 in the placenta, umbilical cord, and decidua. Therefore, it can be concluded that SARS-CoV-2 can penetrate into the placental tissues, can cause an inflammatory reaction, as well as can transmit to the fetus [74]. To correctly diagnose COVID-19 in a child, apart from obtaining a nasopharyngeal smear, chest imaging and rectal swab can be performed [75]. In the case report, Mohakund et al. [76] confirmed COVID-19 infection in a newborn based on a sample collected at 12 h of life. The newborn required resuscitation and mechanical ventilation, but no changes were found on the chest X-ray. Before delivery, COVID-19 infection was also confirmed in the mother of the baby [76]. Moreover, in the case of intrauterine viral transmission from mother to child, attention should be paid to the level of IgM for SARS-CoV-2 and the cytokine IL-6 in the serum of newborns [77]. Dong et al. [78] reported that high levels of IgM were found for SARS-CoV-2 as well as IL-6 and IL-10 in a neonate at 2 h of life, while the nasopharyngeal swab was tested negative. Infection during delivery cannot be excluded; however, IgM antibodies usually appear 3–7 days after infection, and in the reported case, higher levels of IgM antibodies were found in the blood sample taken 2 h after birth. In addition, it should be noted that IgG but not IgM antibodies can be transmitted to the fetus via the placenta. Therefore, the increased levels of IgM antibodies suggest that the newborn may have been infected during pregnancy. RT-PCR tests were not performed for the amniotic fluid and placental sample in the study [71]. A similar result was obtained in the study of Zeng et al. [79], in which none of the six tested newborns were diagnosed with COVID-19 infection when nasopharyngeal swabs were examined. However, in two of them, increased levels of IgG and IgM were found, while in three, increased levels of only IgG were found [79]. Fenizia et al. [80] detected the SARS-CoV-2 genome in 2 maternal plasma attempts. These women had severe clinical outcome. In one sample, they investigated the SARS-CoV-2 genome in placental tissue, vaginal swabs, and cord plasma. However, SARS-CoV-2 IgM were detected in 32% of the maternal plasma and in only one newborn cord plasma, while SARS-CoV-2 IgG presented in 63% of the maternal plasma and 40% of the umbilical cord plasma [80].

The results of many studies on the mother-to-child transmission of SARS-CoV-2 are inconclusive; nevertheless, they indicate the possibility of maternal–fetal transmission. Vertical transmission of SARS-CoV-2 is possible but is rare [64]. A newborn baby is typically infected by the mother or other caregiver with COVID-19 via the droplet route [81]. Therefore, it is necessary to conduct further research on the transmission of virus on the mother–child line.

The studies that analyzed the mother-to-child transmission of SARS-CoV-2 are presented in Table 2.

### 1.5. Management of a Pregnant Woman Infected with COVID-19

Maternal SARS-CoV-2 infection is not an indication of an earlier term of labor or a cesarean section. The health condition of a pregnant woman as well as the fetus should be carefully assessed by a multidisciplinary team [85]. Premature delivery and cesarean section are indicated for pregnant women infected with COVID-19 in the event of severe or critical symptoms (e.g., pneumonia, worsening dyspnea, respiratory failure resulting in the need for mechanical ventilation, and failure of other organs) [54,86].

According to the guidelines of the Centers for Disease Control and Prevention (CDC), pregnant women admitted to hospital with suspected or symptomatic COVID-19 infection should be prioritized to undergo appropriate diagnostic tests. In turn, the examination of asymptomatic women depends on the doctor and medical facility [81]. The way in which a pregnant woman is cared for depends on the country’s health care system. Isolation is of particular importance in dealing with these women regardless of the system [87]. It is recommended that delivery should be carried out in a negative-pressure isolation ward, and the medical staff should use protective clothing throughout the labor [86].

During the hospitalization of pregnant women, visits should be limited to people who are necessary to ensure their well-being and care [87]. All newborns born to women with confirmed or suspected SARS-CoV-2 infection should be tested for COVID-19, regardless of whether they show signs of infection. Neonates should be tested by an RT-PCR test from nasopharyngeal, oropharyngeal, or nasal swabs. If the mother has COVID-19 or there is a risk of infection, tests on the newborn (regardless of the appearance of symptoms) should be performed within 24 h of life. If the result is negative or unavailable, the test should be repeated in at 48 h of life. In places with limited testing capabilities, priority is given to newborns with COVID-19 symptoms and those with exposure to SARS-CoV-2. However, it should be noted that testing too early may result in false-positive results (e.g., part of the neonatal nasopharynx is contaminated with SARS-CoV-2 RNA from maternal fluids) as well as false-negative results—viral RNA may not be detectable immediately after delivery [81].

The guidelines of the Royal College of Obstetricians and Gynaecologists (RCOG) indicate that every woman suspected of COVID-19 must be treated as infected until the test result is obtained [88]. The guidelines of the ACOG (American College of Obstetricians and Gynecologists) highlight that mothers with suspected or confirmed COVID-19 should be separated from others. Although separation of the newborn from the mother reduces its risk of infection, this may result in excessive stress as well as the interruption of breastfeeding. Infants born to women diagnosed with COVID-19 at delivery are recognized and treated as suspected of being infected (tested for SARS-CoV-2, isolated from healthy infants). However, those infants born to women who were not tested for SARS-CoV-2 or for whom the test result is unknown are treated as unsuspected [89]. The CDC recommends that a woman who is infected or suspected of COVID-19 should discuss (preferably during prenatal care) with healthcare professionals whether she would like the newborn baby to be in a room with her or be isolated. The risk of infection of the newborn from the mother does not exist when at least 10 days have passed since the first symptoms or 20 days after the critical course of the disease. In addition, the risk is low when 24 h have passed since the last fever (and no medications were taken) and other symptoms have improved. Separation is necessary for newborns from the high-risk group and when the mother is too ill to care for their infants or need higher levels of care. Nevertheless, isolation of the mother (infected or suspected with COVID-19) is not necessary when the infant is infected with SARS-CoV-2 [82]. The risk of infection of the newborn from the mother is low when she follows hygiene guidelines (mask and washing hands) [90].

There are also recommendations for family births. Before delivery, all birth partners should be asked if they have had symptoms of COVID-19 infection in the last 14 days. In a situation where symptoms have appeared in the last 10 days and the partner has not tested for COVID-19, they must immediately leave the maternity ward. Regardless of the test result, if the partner suffers from a fever within 48 h, they should leave the ward immediately. In both cases, self-isolation at home is recommended [88].

### 1.6. Treatment Options for Pregnant Women Infected with COVID-19

In pregnant women with suspected/confirmed SARS-CoV-2 infection whose clinical condition is deteriorating, respiratory rate as well as oxygen saturation should be monitored. Through appropriately adjusted oxygen therapy, oxygen saturation should be kept above 94% [88]. Depending on the severity of the disease, oxygen supplementation may be performed through the nasal cannula. However, intubation, mechanical ventilation, or extra-corporal membrane oxygenation may also be necessary [17]. In the case of pregnant women suffering from COVID-19, drugs should be selected by interdisciplinary medical personnel, taking into account the safety of the drug for the pregnant woman and the fetus [91].

According to the initial recommendations of ACOG (American College of Obstetricians and Gynecologists), antenatal corticosteroids were allowed to be continued in the preterm period for women with suspected or confirmed COVID-19 but were recommended to be discontinued for pregnant women with suspected or confirmed COVID-19 at 34 0/7–36 6/7 weeks of gestation [92] Only pregnant women who receive oxygen therapy (SpO_2_ values below 94%) or are mechanically ventilated should receive steroid therapy [93]. ACOG recommends treatment with dexamethasone in pregnant women suffering from COVID-19 [92]. During pregnancy, a four-dose course of dexamethasone over 2 days can be used. Thereafter, dexamethasone should be replaced with methylprednisolone. Currently, there is limited information on the effects of dexamethasone on the postpartum period and breastfeeding (methylprednisolone is suggested). However, if a woman is not breastfeeding, dexamethasone may be used [93].

Another drug considered to use in COVID-19 therapy are antiviral drugs and, among them, remdesivir. On 22 October 2020, the FDA (Food and Drug Administration) approved remdesivir for the treatment of specific cases of COVID-19 [94], what is in line with NIH (National Institutes of Health) recommendation on 3 November 2020 [91]. Nevertheless, the most recent studies conducted by the WHO Solidarity trial consortium [95] and made available in pre-review form on 25 October should be taken into account. It has been shown that remdesivir does not or only slightly reduces the risk of death in COVID-19 patients and does not shorten their hospital stay [95]. Other antiviral drugs such as chloroquine, hydroxychloroquine, and lopinavir/ritonavir are also considered in the treatment of COVID-19 in pregnant women [96,97]. Nevertheless, it should be noted that, currently, none of them has received the NIH recommendation for treatment of COVID-19 patients [91]. This applies to both chloroquine or hydroxychloroquine combined with or without azithromycin as well as lopinavir/ritonavir and ivermectin. Another drug proposed to use in COVID-19 therapy is interferon (IFN-a/β) [98,99]. Nevertheless, recent WHO studies show that interferon, hydroxychloroquine, and lopinavir do not improve the condition of COVID-19 patients [91]. In the treatment of COVID-19 patients can be also considered tocilizumab. In a case report by Naqvi et al., pregnant woman with COVID-19 were treated with tocilizumab and remdesivir. It has been reported that these drugs may be effective in treating pregnant women infected with SARS-CoV-2, but more research is needed [100].

Currently, in most clinical trials of drug therapy in COVID-19, pregnant women are excluded. This limits the formulation of appropriate recommendations for the treatment of pregnant women with SARS-CoV-2 infection [91]. At present, only these two drugs have been recommended.

Moreover, pregnancy increases the risk of thrombosis, which is why some authors recommend the use of anticoagulant prophylaxis in patients with COVID-19 [101,102]. The decision on the initiation and duration of prophylactic anticoagulation therapy in pregnant women with COVID-19 should depend on four basic principles: disease severity; hospitalization/home isolation; the presence of the disease and the time remaining until delivery; and the prothrombotic risk associated with comorbidities, pregnancy, postpartum complications, and COVID-19 [103]. In pregnant and puerperal women, unfractionated heparin, low molecular weight heparin, and warfarin are recommended and can also be used safely during breastfeeding [91].

### 1.7. Breastfeeding in the COVID-19 Era

The WHO has recognized that mother’s milk is the basic diet necessary for the proper development and health of infants, young children, and mothers as well. It recommends that babies should be exclusively breastfed for the first 6 months of life while feeding with complementary foods may be continued for 2 years or longer [104]. The CDC guidelines emphasize that proper hygiene should be maintained during feeding, while the United Nations Children’s Fund (UNICEF) has defined these recommendations as the “3W” principle (wear a mask while feeding, wash hands with soap before and after touching the baby, and wipe and disinfect surfaces) [105,106]. The mother, along with her family and medical staff should make a decision about whether to start or continue breastfeeding. She should also maintain adequate personal hygiene before and while expressing breast milk either manually or using a breast pump [105]. Women who, due to the severity of the disease, are unable to feed their children on their own should consult a doctor. In such a situation, it is necessary to obey the 3W rules. The mother can pump milk, while an uninfected family member should feed the newborn [106]. Most of the available studies do not confirm that SARS-CoV-2 may be transmitted to infants via breast milk [10,107,108]. However, Wu et al. [109] analyzed a sample of milk collected from a patient 1 day after delivery and observed the presence of SARS-CoV-2. A similar result was not found in the milk sample taken on the 3rd day after delivery as well as in the milk obtained from the other examined patients [109]. The report of Hinojos-Velasco et al. [59] provided evidence of the potential risk of infecting the newborn with SARS-CoV-2 both during pregnancy and breastfeeding. A 21-year-old pregnant woman was admitted to the maternity ward with symptoms of COVID-19. Due to severe symptoms and confirmation of the infection with RT-PCR, an urgent caesarean section was performed. The newborn was separated from the mother immediately after birth (no skin-to-skin contact). Nevertheless, the collected during labor swabs from the nasopharyngeal and oropharyngeal confirmed infection of COVID-19 in the neonate. On the fourth postpartum day, SARS-CoV-2 RNA was found in the mother’s milk and stool samples as well as in the baby’s stool. On the 13th after birth, negative results were obtained from the newborn [59]. The presence of SARS-CoV-2 in milk was also demonstrated by Bastug et al. [58]. The milk sample was tested after the first lactation and on the 3rd and 4th days after delivery. All tests gave a positive result. In turn, in the first test performed on a newborn 8 h after birth, the result was negative; however, on day 4, SARS-CoV-2 infection was confirmed in a swab, stool, and blood. Nevertheless, due to the asymptomatic form of COVID-19 in both mother and child, the mother was encouraged to breastfeed her baby on day 5 after delivery [58]. In turn, Yu et al. [110] presents the case of an infected mother and a 13-month-old child. The woman was constantly breastfeeding the child. Both cases were probably infected during a family gathering. Nevertheless, a milk sample was taken for the tests. The milk showed negative results for the presence of SARS-CoV-2; however, on the 9th and 25th days of hospitalization, the results were positive for the presence of IgG antibodies and negative for IgM [110].

The WHO also recommends that mothers with suspected or confirmed COVID-19 infection can breastfeed their infants. Moreover, regardless of whether the mother or infant has been diagnosed or is suspected of COVID-19, a mother and her child should be allowed to stay together in a room [105].

### 1.8. Emotional State of Pregnant Women During the COVID-19 Pandemic

COVID-19 may increase the levels of anxiety, insecurity, and stress among women both during pregnancy and after childbirth [111]. A significantly higher rate of depressive symptoms was noted in pregnant women after announcement of the COVID-19 epidemic than before. In addition, after the COVID-19 outbreak, pregnant women were reported to have frequently thought about self-harm. It should be noted that the depressive indicators were positively related to the number of COVID-19 cases confirmed every day and the number of suspected infections and deaths [112]. Saccone et al. [113] showed that the level of anxiety was higher among women during the first trimester of pregnancy compared to the second and third trimesters [113]. In turn, Qiu Ju Ng et al. [114] observed that women who believed that SARS-CoV-2 infection could be transmitted to the developing child during pregnancy had significantly higher rates of anxiety.

The main reason provided for the increased level of anxiety was the fear of possible intrauterine transmission of COVID-19 to the fetus [113]. COVID-19 has also increased the level of depression as well as general and specific anxiety among pregnant women [115]. Moreover, a study in Italy showed that women who gave birth during the compulsory quarantine period had a significantly higher level of depressive disorders compared to those who gave birth in the same period in 2019 [116]. However, in China, it was observed that nonpregnant women had a higher level of depression and anxiety compared to pregnant women (17.5% vs. 5.3% and 17.5% vs. 6.8%, respectively) [117]. In turn, in studies conducted among pregnant Iranian women, symptoms of depression were diagnosed in 32.7% of them. Pregnant women also had high levels of stress (32.7%) and anxiety (43.9%) [118]. It should be mentioned that an incorrect psychosocial state in pregnant women may have negative effects on the health of both the mother and the child (e.g., gestational hypertension, premature delivery, weakening of the fetal immune system, and impaired cognitive development of the child) [114,119]. Therefore, it is necessary to support women in the perinatal period [120,121]. Studies that examined the emotional state of pregnant women during the COVID-19 pandemic are presented in Table 3.

## 2. Conclusions

Similar to the other groups of patients, the most important factor determining the course of COVID-19 in pregnant women is the age and presence of comorbidities, such as gestational hypertension, gestational diabetes, and cholestasis, mainly in the second and third trimesters of pregnancy. COVID-19 has not been confirmed to increase the risk of infection and death among pregnant women; however, pregnant women had increased risk of developing more severe symptoms. The part of currently considered optimal diagnostics (X-ray and CT) and treatment (antibiotic therapy and immunotherapy) of the disease cannot be applied to pregnant women due to their harmfulness to the fetus and feeding in the postpartum period. This may delay diagnostic and therapeutic procedures in the case of pregnant women, especially those with more severe symptoms. Screening tests are important for pregnant women because of the possibility of vertical infection of the fetus, which can be avoided by knowing the mother’s immune status. Still, a small number of scientific reports do not allow for full assessment of the risk and consequences of SARS-CoV-2 infection for pregnant women and neonates. Therefore, it is necessary to conduct further observations and, based on them, to draw conclusions or recommendations that may increase the safety of both the mother and the fetus or her newborn baby.

## Figures and Tables

**Table 1:** Symptoms of Coronavirus Disease 2019 (COVID-19) among pregnant women in prospective, retrospective and case studies.

**Author, Country**	**Design**		**Objective**		**Materials and Methods**		**Results**
Khoury, United States [42]	Prospective cohort study		To describe the characteristics and birth outcomes of women with COVID-19		A total of 241 pregnant women * (RT-PCR performed on an NPS before/during delivery hospitalization) were recruited from 13 March to 12 April 2020. Follow up was on 20 April 2020. Infants (n = 236) were tested by RT-PCR on an NPS at 24 h of life and repeated until 96 h of life.		At the time of admission, 61.4% of women were asymptomatic for COVID-19. During delivery, 26.5% of women had mild symptoms, 26.1% had severe symptoms, and 5% had critical symptoms. Of those with severe and critical COVID-19, 52.4% and 91.7%, respectively, underwent cesarean delivery. There were no maternal deaths. Among the infants, 97.5% were negative for SARS-CoV-2.
Liu, China [47]	Retrospective study		Clinical characteristics of pregnant women diagnosed with COVID-19		A total of 13 pregnant women * (age: 22–36 years) (RT-PCR performed on an OPS) were hospitalized between 8 December 2019 and 25 February 2020 Gestational age: 25–36 wk		Caesarean section was performed in 10 women (77%), including 5 emergency sections due to pregnancy complications: fetal distress, stillbirth, or premature rupture of the membrane, with preterm labor (32–36 wk) in 6 cases (46%). One woman developed critical pneumonia with MODS (7.6%). No vertical transmission was found.
Yu, China [53]	Retrospective, single-center study		To clarify the clinical features as well as obstetric and neonatal outcomes of pregnant patients with COVID-19		Seven pregnant women * (RT-PCR performed on throat swab specimens from the upper respiratory), with a mean age of 32 years (29–34), from 1 January to 8 February 2020, presented with fever (6), cough (1), shortness of breath (1), and diarrhea (1); 100% of births were cesarean section. Three infants were tested by RT-PCR from throat swabs.		The clinical characteristics of pregnant patients with COVID-19 were similar to those of nonpregnant women with COVID-19. The outcomes of the pregnant women and neonates were good. Three neonates were tested negative for SARS-CoV-2, while one was infected with SARS-CoV-2 36 h after birth.
Ferrazzi, Italy [54]	Retrospective study		To report the mode of delivery and immediate neonatal outcome in women with COVID-19		Forty-two women * with COVID-19 (RT-PCR performed on NPS) delivered during 1–20 March 2020. The diagnosis of COVID-19 in newborns was made on the basis of NPS.		Delivered vaginally: 24; cesarean section: 18. Outcomes in women: pneumonia (19) and oxygen support (7). Four women were admitted to the ICU. Two infants were tested positive for COVID-19 at days 1 and 3 (breastfeed without surgical mask). One newborn had a positive test after an operative vaginal delivery (separated from mother immediately after birth).
Brandt, USA [15]	A matched case-control study		The impact of COVID-19 on adverse outcomes in pregnant women and newborns (epidemiology and risk factors)		With delivery between 11 March and 11 June 2020, 183 women were divided into two groups in a 2:1 ratio. Women who were not infected with SARS-CoV-2 were assigned to the control group (n = 122), while women with confirmed COVID-19 * (nasopharyngeal swab) were enrolled to the COVID-19 cases group (n = 61).		Mild COVID-19 disease: 88.5%, severe: 9.8%, and critical: 1.6%. Mothers suffering from COVID-19 had a 3.4 times greater chance of developing adverse maternal outcomes than mothers in the control group (18.0% vs. 8.2%; adjusted odds ratio, 3.4; 95% CI 1.2–13.4). It has been reported that newborns born to mothers with COVID-19 had a 1.7 times greater chance of obtaining unfavorable neonatal outcomes compared to infants from the control group (18.0% vs 13.9%; adjusted odds ratio, 1.7; 95% CI 0.8–4.8).
**Author, Country**		**Design**		**Objective**		**Case Characteristics**	
Richtmann, Brazil [51]		Case study		To describe the cases of five pregnant women		Five pregnant women had mild or moderate COVID-19 symptoms. Each woman was obese or overweight, while none of them had any known comorbidities that could affect the pregnancy. Patient 1 reported to the hospital at 27 weeks of gestation with rhinorrhea, myalgia, and fever for the last 3 days and shortness of breath in the last 24 h. Patient 2 reported to the hospital in the 21st week of pregnancy with fever and cough for the last 6 days. Patient 3 was asymptomatic and presented to the hospital at 38 weeks of pregnancy due to reduced baby movements. Patient 4, who was 23 weeks pregnant, came to the hospital with pain in the lower abdomen. Patient 5 presented at 30 weeks of gestation with rhinorrhea, fever, headache, anosmia, and dysgeusia starting 6 days earlier. Fetal death occurred at 21–38 weeks of gestation. In all cases of mothers, COVID-19 was confirmed by an RT-PCR test of NPS. CT values below 33 were noticed positive. For SARS-CoV-2 RT-PCR testing, placental fragments (5 mm^3^) were collected immediately after delivery. Amniotic fluid samples (≥2 mL) were also collected during labor. One newborn tested positive for SARS-CoV-2 in the placenta, while the other tested positive for both the placenta and the amniotic fluid.	
Li, China [43]		Case study		To describe the critical case of COVID-19 in a pregnant woman		The patient presented to the hospital at 35 weeks of pregnancy. The first symptoms of COVID-19 were sore throat, dry cough, fever, and dyspnea. Due to the rapid worsening of symptoms (from dyspnea to acute respiratory distress syndrome and septic shock within 12 h), a cesarean section was performed, but unfortunately, the newborn died within 2 h of birth. The disease also led to disorders in the heart, kidneys, and liver of the mother. In the mother, SARS-CoV-2 infection was confirmed by RT-PCR test.	
Alzamora, Peru [44]		Case study		To describe the case of a pregnant patient with COVID-19 and the newborn positive for SARS-CoV-2		A pregnant woman with COVID-19 (RT-PCR test was done from NPS), aged 41, who was at 33 weeks of gestation, reported with respiratory insufficiency and diabetes mellitus and required mechanical ventilation. The patient underwent a cesarean section, and the neonate’s Apgar scores were 6 (1 min) and 8 (5 min). There was no delayed cord clamping or skin-to-skin contact. Breastfeeding was not initiated. After 16 h of birth, the NPS of neonate was tested positive for SARS-CoV-2. The newborn required ventilatory support for 12 h and did not required antibiotic treatment. The newborn was serology negative for IgG and IgM; maternal serology was negative on postpartum day 1 but positive on postpartum day 4.	
Reis, Brazil [55]		Case study		To describe the cases of three pregnant women with COVID-19 and their newborns		Patient 1: Aged 28; had fever for 3 days and abdominal pain; underwent delivery by cesarean section and had preterm birth. Infant’s RT-PCR result for COVID-19 was negative. After 23 days, the woman was discharged. Patient 2: Aged 34; had fever for 4 days, cough, and shortness of breath; underwent delivery by cesarean section and had term birth. Infant’s RT-PCR result for COVID-19 was negative. After being in the ICU for 13 days, the woman was transferred to rehabilitation ward. Patient 3: Aged 25; had fever for 14 days, cough, shortness of breath, diarrhea, and abdominal pain; underwent delivery by cesarean section and had preterm birth. Infant’s RT-PCR result for COVID-19 was negative. After being in the ICU for 22 days, the woman died. All women had SARS-CoV-2 infection confirmed by an RT-PCR test performed on NPS. All 3 newborns were tested negative for RT-PCR from the NPS.	
Dong, China [56]		Case study		To detect the antibodies and SARS-CoV-2 in the breast milk of a maternal woman with COVID-19		A 33-year-old woman (38 weeks and 2 days of gestation) was confirmed positive for SARS-CoV-2 through RT-PCR from throat swabs (a Ct-value < 40 was noticed as a positive result). The woman had mild symptoms and positive radiologic imaging findings and gave birth in a negative-pressure operating room. The infant was negative for SARS-CoV-2 (sample from OPS). The virus was not detected in the mother’s body fluids (breast milk, urine, vaginal secretion, feces, tear, sweat, and blood) after delivery despite positive results for SARS-CoV-2 in throat swabs. IgA and IgG antibodies were detected in breast milk, which demonstrated potential immune protection for the neonate.	
Lowe, Australia [57]		Case study		To describe the case of a woman with COVID-19 who had vaginal delivery		A 31-year-old woman with COVID-19 spontaneously labored. The woman had a temperature of 38.4 °C intrapartum with ongoing respiratory symptoms. Respiratory saturations remained normal throughout labor. Staff wore full personal protective equipment, and the patient wore a surgical mask during the second stage of labor. There was no maternal–neonatal separation. The neonate was tested negative for COVID-19.	
Vallejo, United States [45]		Case study		To describe the fatal case of a pregnant woman with COVID-19		A 36-year-old patient (37 weeks of gestation) suffered from shortness of breath, fever, cough, and sore throat. After admission, the patient experienced respiratory distress, needed intubation, and underwent cesarean delivery. In the ICU, the patient subsequently decompensated. Despite supportive measures, the patient had multiorgan failure, sepsis, and cardiopulmonary arrest within 36 h. An NPS (RT-PCR test) was performed to diagnose COVID-19. The newborn had a negative RT-PCR result, and no pathological changes were found in the placenta.	
Bastug, Turkey [58]		Case study		To report the presence of SARS-CoV-2 in breast milk		A 20-year-old pregnant woman with no COVID-19 symptoms was tested for SARS-CoV-2 and was positive (RT-PCR test from NPS, Ct value 31.26). After vaginal delivery, the baby was separated. The infant was negative for SARS-CoV-2 (8–10 h after birth, RT-PCR test). The infant was fed with breast milk for 24–36 h. After first lactation, a breast milk sample was tested for SARS-CoV-2 (RT-PCR test) and was positive (the Ct values determined within 3 days were 29.20, 28.85, and 32.28). At 96 h, the infant’s NPS, blood, and stool were tested for SARS-CoV-2 and all were positive (RT-PCR test, Ct values for NPS, 32.71; blood, 33.10; and stool, 32.84).	
Hinojosa-Velaso, Mexico [59]		Case study		To describe infant and mother with severe COVID-19 and to detect SARS-CoV-2 in samples		A 21-year-old pregnant women with COVID-19 symptoms (coughing, odynophagia, headache, diarrhea, and rhinorrhea) was positive for SARS-CoV-2 (RT-PCR test was performed on the basis of NPS and OPS; a Ct-value < 40 was noticed as a positive result). She had a caesarian section. The neonate had tachypnea, hyponatremia, central cyanosis, dyspnea and oxygen saturation—87%. Mother had severe respiratory depression and needed intubation. Mother’s milk and stool samples were positive for SARS-CoV-2. NPS and OPS samples were taken from the newborn during delivery—the RT-PCR test confirmed SARS-CoV-2 infection with a low threshold value—suggesting high viral load. The newborn’s stool was also positive for SARS-CoV-2 at the 5th day after birth, but negative at the 13th day of life.	

RT-PCR—real-time reverse transcription polymerase chain reaction; * with laboratory-confirmed SARS-CoV-2 in the RT-PCR technique; ICU—Intensive Care Unit; MODS—multiple organ dysfunction syndrome; NPS—nasopharyngeal swab; OPS—oropharyngeal swabs; wk—week.

**Table 2:** Characteristics of studies showing intrauterine transmission from mother to child.

Author, Country	Mothers/Neonates	Mode of Delivery	Symptoms of COVID-19 in Neonates	Samples and Time of Sample Collection from Neonates	Sample Test Results of Neonates
Alzamora, Peru [44]	1/1	CD: 1	Due to the high level of maternal sedation, the newborn was intubated. At 6 days after delivery, the newborn presented mild respiratory problems and cough.	At birth, a test of serum sample IgG and IgM was conducted; NPS 16 h after delivery and 48 h later (RT-PCR test) were conducted.	The result of serum sample IgG and IgM was negative, and that of NPS was positive.
Wang, China [66]	1/1	CD: 1	Changes in chest radiograph	A pharyngeal swab from the newborn after 36 h of birth and from the mother and the mother’s breast milk sample, cord blood, and placental sample were tests for SARS-CoV-2 (RT-PCR).	Pharyngeal swab was positive for mother and newborn at 36 h of birth; breast milk, cord blood, and placental samples were negative. Fifteen days after birth newborn’s pharyngeal and anal swab were negative in RT-PCR.
Vivanti, France [69]	1/1	CD: 1	The infant needed active resuscitation after birth; after 6 h, the baby was extubated. On the 2nd day of life, the newborn showed irritability, axial hypertonia, and opisthotonos. In the following days, the condition of the newborn improved.	A RT-PCR test from the neonate sample of NPS (1 h, 2 days, and 18 days of life), rectal swab, and blood was conducted. The mother was tested from NPS, blood, vaginal swab, placenta, and amniotic fluid. Ct value < 40 was interpreted as positive for SARS-CoV-2.	Neonate’s RT-PCR test results (vl): blood—1.15; NPS (1 h)—2.21, (3 days)—7.3, (18 days)—4.54; and rectal swab—4.71. Mother’s RT-PCR test results (vl): NPS—4.22; vaginal swab—0.63; placenta—11.15; amniotic fluid—2.09; and blood—4.87.
Patanè, Italy [71]	22/2	VD: 1; CD: 1	In both cases, there were no symptoms of COVID-19.	All mothers (22) and neonates (22) were tested by RT-PCR test from NPS. Placental samples were also tested.	NN1: positive at 24 h and 7days of birth; NN2: negative at birth and positive after 7days. In both cases, placenta showed chronic intervillositis, with the presence of macrophages.
Zeng, China [79]	33/3	CD: 3	NN1: lethargy, fever, and pneumonia; NN2: lethargy, vomiting, fever, leukocytosis, lymphocytopenia, an elevated creatine kinase-MB fraction, and pneumonia; NN3: neonatal respiratory distress syndrome and pneumonia	NN1–NN2: nasopharyngeal and anal swabs at 2, 4, and 6 days after birth (RT-PCR); NN3: nasopharyngeal and anal swabs at 2, 4, and 7 days after birth (RT-PCR)	In all cases, nasopharyngeal and anal swabs were positive for SARS-CoV-2 on days 2 and 4 after birth. On days 6 (NN1 and NN2) and 7 (NN3), the results for SARS-CoV-2 were negative.
Wu, China [82]	29/2	CD: 2	NN1: ground-glass opacity in chest; NN2: fever and ground-glass opacity in chest	Mothers: RT-PCR test from throat swab; if RT-PCR was not available, diagnosis was made by chest CT scan. Neonates: RT-PCR test from throat and anal swabs, and X-rays/CT scan of chest; in 4 neonates: level of IgG and IgM.	Thirteen mothers were diagnosed with SARS-CoV-2 by RT-PCR test; 16 were diagnosed by chest CT-scan. Positive results on the RT-PCR test from throat swab were found in 2 newborns. Three newborns were diagnosed as suspected of being infected with SARS-CoV-2 (throat swabs were negative, IgG and IgM were positive, and changes were found in chest images).
Hu, China [83]	7/1	CD: 6; VD: 1	None of the infants presented clinical symptoms.	Neonates: NPS, blood, feces, and urine at 24–36 h after birth (RT-PCR) and chest X-ray Mothers: throat swabs (RT-PCR), amniotic fluid (in 7 cases, RT-PCR test), and placenta	One infant was positive for a SARS-CoV-2 RT-PCR test in NPS at 36 h of life. Blood, urine, and feces samples were negative; chest X-ray was normal. Mothers: each woman had a positive throat swab from the RT-PCR result; all results from amniotic fluid were negative; one patient had thickness of fetal membranes; and in other cases, placenta were normal.
Marzollo, Italy [84]	1/1	VD:1	The infant showed feeding intolerance, abdominal distension, and hyporectivity.	Mother: NPS by RT-PCR Newborn: NPS by RT-PCR immediately after birth, NPS at 36 h of life and 17 days of life, RT-PCR from tracheal aspiration, RT-PCT from anal swab at 19 days of life, blood, and chest X-ray. RT-PCR from NPS was repeated one month after delivery.	Mother: positive results of RT-PCR test by NPS sample. Newborn: immediately after birth, the result of RT-PCR was inconclusive, the result was positive at 36 h of life, and the results was also positive from tracheal aspiration. At 17 days of life, the newborn had positive results from NPS and an anal swab (RT-PCR). After one month from delivery, the newborn had negative results from NPS. Chest X-ray showed mild changes.
Mohakud, India [76]	1/1	CD:1	The infant required resuscitation and mechanical ventilation immediately after delivery.	Mother: NPS by RT-PCR Newborn: tracheal aspirate at 12h of life and chest X-ray	Mother: positive results at RT-PCR from NPS. Newborn: The X-ray showed no changes in the chest area. After 12 h of life, a sample of tracheal aspirate was taken and a positive result for COVID-19 was obtained.
Fenizia, Italy [80]	30/2	CD:6 VD: 25	The infants were totally asymptomatic.	Mothers: RT-PCR from NPS, vaginal swabs, placenta, amniotic fluids, umbilical cord plasma and umbilical cord, and milk Neonates: NPS from RT-PCR, Viral RNA, IgM, IgG; Ct-value <40 was defined as a positive test result	Mother: 30/31 had positive results from NPS. Neonates: 2 had positive results from NPS. Viral RNA was in the following samples: maternal plasma—2; vaginal swabs—1; placenta—1; umbilical cord plasma—1; umbilical cord—0; amniotic fluid—0; and milk—1. In maternal plasma, umbilical cord plasma, and milk the presence of IgG/IgM antibodies was reported.
Dhawan, India [67]	1/1	CD:1	A newborn was born on 34 + 4 weeks; the premature baby developed respiratory distress.	Mother: throat swab (RT-PCR) Newborn: NPS at RT-PCR and chest X-ray	Mother: positive result at throat swab Newborn: RT-PCR was positive at 23 h of the newborn’s life; chest X-rays were normal; and the newborn was discharged home on day 7 with negative results.
Sisman, USA [68]	1/1	VD:1	A newborn was born on 34 weeks; on the 2nd day after delivery, the newborn developed fever, respiratory distress, and hypoxia. Respiratory symptoms disappeared on day 3rd of their onset.	Mother: NPS (RT-PCR) Newborn: NPS at RT-PCR, blood count, chest X-ray, and placenta histopathology	Mother: positive result at NPS. Newborn: RT-PCR was positive at 24 h, 48 h, and 14 days of the newborn’s life; chest X-ray was normal; and placenta histopathology revealed SARSCoV-2 infection.

RT-PCR—real-time reverse transcription polymerase chain reaction; *—with laboratory-confirmed SARS-CoV-2 in the RT-PCR technique; VD—vaginal delivery; CD—cesarean delivery; NN—neonate number; CT—computed tomography; NPS—nasopharyngeal swab; vl—viral load.

**Table 3:** Emotional state of pregnant women during the COVID-19 pandemic.

Ref.	Design	Objective	Materials and Methods	Results
Wu, China [112]	Cross-sectional study, multicenter	To examine the impact of COVID-19 on the prevalence of depressive symptoms and anxiety among pregnant women across China	G.1: 2839 pregnant women, recruited from 1 January to 20 January G.2: 1285 pregnant women, recruited from 21 January to 8 February (after the declaration of the COVID-19 epidemic); assessment: EPDS	Pregnant women from G.2. had significantly higher rates of depressive symptoms (*p* = 0.02) and more likely had thoughts of self-harm (*p* = 0.005). The depressive rates were associated with the number of COVID-2019 cases, suspected infections, and deaths.
Saccone, Italy [113]	Cross-sectional study	To evaluate the psychological impact and anxiety during the COVID-19 pandemic	One hundred pregnant women (17 in the first, 35 in the second, and 48 in the third trimester). Assessment: IES-R to measure the psychological impact of COVID-19 and six-item short form of the state scale of STAI and VAS to measure anxiety due to COVID-19	Most women rated the psychological impact of the COVID-19 outbreak as severe and reported high anxiety regarding the vertical transmission of the disease. The anxiety and psychological impact were more among women who were in the first trimester of pregnancy.
Zanardo, Italy [116]	Nonconcurrent case–control study	To explore whether quarantine and hospital policies increased psychoemotional distress among women in the early postpartum period who were giving birth in a COVID-19 area in Italy	G.1: (COVID-19) women aged over 18, delivery between 8 March 8 and 3 May 2020 G.2: (control) women aged over 18, lived in the same geographic area, and delivered from March to May 2019 Assessment: EPDS	The COVID-19 study group had significantly higher mean EPDS scores (*p* < 0.001) and higher scores for anhedonia and depression. Concerns about the risk of COVID-19 and quarantine measures during the COVID-19 pandemic affected the emotions of mothers, worsening their depressive symptoms.
Qiu Ju Ng, China [114]	Cross-sectional study	Assessment of knowledge about COVID-19 and the levels of depression, stress, and anxiety in pregnant women	324 pregnant women, From 31 March to 25 April, assessment: DASS-21, a survey about women’s knowledge of COVID-19	Women who believed that the SARS-CoV-2 could be transmitted to the baby during pregnancy and could cause fetal anomalies and intrauterine death had elevated levels of anxiety.
Effati-Daryani, Iran [118]	Cross-sectional study	The levels of depression, stress, and anxiety and their predictors in Iranian pregnant women during the COVID-19 pandemic	205 pregnant women, examined from of March to April 2020, no one had COVID-19 Assessment: DASS-21, sociodemographic questionnaire	Marital life satisfaction, a high level of spousal education, and income were associated with reduced symptoms of stress, anxiety, and depression in pregnant women.
Zhou, China [117]	Cross-sectional study	Assessment of depression in pregnant women and comparison of the emotional level with nonpregnant women	544 pregnant and 315 nonpregnant women from 41 cities of China, they were examined from 28 February to 12 March 2020 Assessment: PHQ-9, GAD-7, ISI, SCL-90, PCL-5	During the pandemic, pregnant women had lower levels of depression, anxiety, insomnia, and PTSD compared to women at reproductive age but who are not currently pregnant

EPDS—Edinburgh Postnatal Depression Scale; IES-R—Impact of Event Scale-Revised; STAI—Spielberger State-Trait Anxiety Inventory; VAS—Visual Analog Scale; DASS-21—Depression, Anxiety and Stress Scale—21; PHQ-9—The Patient Health Questionnaire; GAD-7—General Anxiety Disorders-7; ISI—Insomnia Severity Index; SCL-90—The Symptom Chechlist-90; PCL-5—Post-traumatic Stress Disorder Checklist-5; PTSD—Post-traumatic stress disorder.

## References

[1] Di Gennaro F., Pizzol D., Marotta C., Antunes M., Racalbuto V., Veronese N., Smith L. (2020). Coronavirus Diseases (COVID-19) Current Status and Future Perspectives: A Narrative Review. Int. J. Environ. Res. Public Health.

[2] Khadka R., Bhandari R., Gyawali R., Neupane B., Pant D. (2020). Epidemiology and Pathogenesis of Coronavirus Disease (COVID-19). NRMJ.

[3] Anjorin A.A. (2020). The coronavirus disease 2019 (COVID-19) pandemic: A review and an update on cases in Africa. Asian Pac. J. Trop. Med..

[4] World Health Organization Coronavirus Disease (COVID-19) Pandemic. https://www.who.int/emergencies/diseases/novel-coronavirus-2019.

[5] Qiancheng X., Jian S., Lingling P., Lei H., Xiaogan J., Weihua L., Gang Y., Shirong L., Zhen W., GuoPing X. (2020). Coronavirus disease 2019 in pregnancy. Int. J. Infect. Dis..

[6] Ryan G.A., Purandare N.C., McAuliffe F.M., Hod M., Purandare C.N. (2020). Clinical update on COVID-19 in pregnancy: A review article. J. Obstet. Gynaecol. Res..

[7] López M., Gonce A., Meler E., Plaza A., Hernández S., Martinez-Portilla R.J., Cobo T., García F., Gómez Roig M.D., Gratacós E. (2020). Coronavirus Disease 2019 in Pregnancy: A Clinical Management Protocol and Considerations for Practice. Fetal. Diagn. Ther..

[8] Allotey J., Stallings E., Bonet M., Yap M., Chatterjee S., Kew T., Debenham L., Llavall A., Dixit A., Zhou D. (2020). Clinical manifestations, risk factors, and maternal and perinatal outcomes of coronavirus disease 2019 in pregnancy: Living systematic review and meta-analysis. BMJ.

[9] Ellington S., Strid P., Tong V.T., Woodworth K., Galang R.R., Zambrano L.D., Nahabedian J., Anderson K., Gilboa S.M. (2020). Characteristics of Women of Reproductive Age with Laboratory-Confirmed SARS-CoV-2 Infection by Pregnancy Status—United States, 22 January–7 June 2020. Morb. Mortal. Wkly. Rep..

[10] Chen H., Guo J., Wang C., Luo F., Yu X., Zhang W., Li J., Zhao D., Xu D., Gong Q. (2020). Clinical characteristics and intrauterine vertical transmission potential of COVID-19 infection in nine pregnant women: A retrospective review of medical records. Lancet.

[11] Khalil A., Kalafat E., Benlioglu C., O’Brien P., Morris E., Draycott T., Thangaratinam S., Le Doare K., Heath P., Ladhani S. (2020). SARS-Cov-2 Infection in Pregnancy: A Systematic Review And Meta-Analysis Of Clinical Features And Pregnancy Outcomes. EClinicalMedicine.

[12] Sutton D., Fuchs K., D’Alton M., Goffman D. (2020). Universal Screening For SARS-Cov-2 In Women Admitted for Delivery. N. Engl. J. Med..

[13] Di Mascio D., Saccone G., Sen C., Galindo A., Grünebaum A., Yoshimatsu J., Stanojevic M., Kurjak A., Chervenak F., The WAPM (The World Association of Perinatal Medicine) Working Group on COVID-19 (2020). Maternal and Perinatal Outcomes of Pregnant Women with SARS-COV-2 infection. Ultrasound Obstet. Gynecol..

[14] Hanna N., Hanna M., Sharma S. (2020). Is pregnancy an immunological contributor to severe or controlled COVID-19 disease?. Am. J. Reprod. Immunol..

[15] Brandt J., Hill J., Reddy A., Schuster M., Patrick H., Rosen T., Sauer M., Boyle C., Ananth C. (2020). Epidemiology of Coronavirus Disease 2019 In Pregnancy: Risk Factors and Associations With Adverse Maternal And Neonatal Outcomes. Am. J. Obstet..

[16] Liu H., Wang L., Zhao S., Kwak-Kim J., Mor G., Liao A. (2020). Why Are Pregnant Women Susceptible To COVID-19? An Immunological Viewpoint. J. Reprod. Immunol..

[17] Liang H., Acharya G. (2020). Novel corona virus disease (COVID-19) in pregnancy: What clinical recommendations to follow?. Acta Obstet. Gynecol. Scand..

[18] Alberca R., Pereira N., Oliveira L., Gozzi-Silva S., Sato M. (2020). Pregnancy, Viral Infection, And COVID-19. Front. Immunol..

[19] Teles Abrao Trad A., Ibirogba E.R., Elrefaei A., Narang K., Tonni G., Picone O., Suy A., Carreras Moratonas E., Kilby M.D., Ruano R. (2020). Complications and outcomes of SARS-CoV-2 in pregnancy: Where and what is the evidence?. Hypertens. Pregnancy.

[20] Lopes de Sousa Á.F., Carvalho H.E.F., Oliveira L.B., Schneider G., Camargo E.L.S., Watanabe E., de Andrade D., Fernandes A.F.C., Mendes I.A.C., Fronteira I. (2020). Effects of COVID-19 Infection during Pregnancy and Neonatal Prognosis: What Is the Evidence?. Int. J. Environ. Res. Public Health.

[21] Kumar A., Beniwal M., Kar P., Sharma J., Murthy N. (2004). Hepatitis E in Pregnancy. Int. J. Gynecol..

[22] Tien Dat T., Kotani T., Yamamoto E., Shibata K., Moriyama Y., Tsuda H., Yamashita M., Kajiyama H., Duc Thien Minh D., Quang Thanh L. (2018). Dengue Fever During Pregnancy. Nagoya J. Med. Sci..

[23] Rasmussen S., Jamieson D., Uyeki T. (2012). Effects of Influenza on Pregnant Women and Infants. Am. J. Obstet. Gynecol..

[24] Lauer S., Grantz K., Bi Q., Jones F., Zheng Q., Meredith H., Azman A., Reich N., Lessler J. (2020). The Incubation Period Of Coronavirus Disease 2019 (COVID-19) From Publicly Reported Confirmed Cases: Estimation And Application. Ann. Intern. Med..

[25] Hoffmann M., Kleine-Weber H., Schroeder S., Krüger N., Herrler T., Erichsen S., Schiergens T., Herrler G., Wu N., Nitsche A. (2020). SARS-Cov-2 Cell Entry Depends on ACE2 And TMPRSS2 And Is Blocked By A Clinically Proven Protease Inhibitor. Cell.

[26] Zou X., Chen K., Zou J., Han P., Hao J., Han Z. (2020). Single-Cell RNA-Seq Data Analysis on The Receptor ACE2 Expression Reveals the Potential Risk Of Different Human Organs Vulnerable To 2019-Ncov Infection. Front. Med..

[27] Chan J., Kok K., Zhu Z., Chu H., To K., Yuan S., Yuen K. (2020). Genomic Characterization Of The 2019 Novel Human-Pathogenic Coronavirus Isolated From A Patient With Atypical Pneumonia After Visiting Wuhan. Emerg. Microbes Infect..

[28] Tufan A., Avanoglu Guler A., Matucci-Cerinic M. (2020). COVID-19, Immune System Response, Hyperinflammation And Repurposing Antirheumatic Drugs. Turk. J. Med. Sci..

[29] Qin C., Zhou L., Hu Z., Zhang S., Yang S., Tao Y., Xie C., Ma K., Shang K., Wang W. (2020). Dysregulation Of Immune Response In Patients With Coronavirus 2019 (COVID-19) In Wuhan, China. Clin. Infect. Dis..

[30] Jørgensen N., Persson G., Hviid T. (2019). The Tolerogenic Function Of Regulatory T Cells In Pregnancy And Cancer. Front. Immunol..

[31] Muyayalo K.P., Huang D.H., Zhao S.J., Xie T., Mor G., Liao A.H. (2020). COVID-19 and Treg/Th17 imbalance: Potential relationship to pregnancy outcomes. Am. J. Reprod. Immunol..

[32] Rasmussen S.A., Smulian J.C., Lednicky J.A., Wen T.S., Jamieson D.J. (2020). Coronavirus Disease 2019 (COVID-19) and pregnancy: What obstetricians need to know. Am. J. Obstet. Gynecol..

[33] Francis S., Mathew R.P., Khalid Z.A. (2020). Coronavirus (COVID-19) Infection in Pregnancy: Does Non-contrast Chest Computed Tomography (CT) Have a Role in Its Evaluation and Management?. J. Obstet. Gynaecol India..

[34] World Health Organization (2020). Laboratory Testing for Coronavirus Disease (COVID-19) in Suspected Human Cases: Interim Guidance. https://www.who.int/publications/i/item/laboratory-testing-for-2019-novel-coronavirus-in-suspected-human-cases-20200117.

[35] Böger B., Fachi M., Vilhena R., Cobre A., Tonin F., Pontarolo R. (2020). Systematic Review with Meta-Analysis Of The Accuracy Of Diagnostic Tests For COVID-19. Am. J. Infect. Control..

[36] Giri B., Pandey S., Shrestha R., Pokharel K., Ligler F., Neupane B. (2020). Review of Analytical Performance Of COVID-19 Detection Methods. Anal. Bioanal. Chem..

[37] Montoya Ramirez E.Y., Gilliot C., Puygrenier M., Kacem R., Schaub M., Homatter C., Amancei S. (2020). Lung Ultrasound for Rapid Diagnosis of COVID-19-Induced Pulmonary Pathology: A Case Report of a Pregnant Infected Woman. Gynecol. Obstet. Case Rep..

[38] Salameh J., Leeflang M., Hooft L., Islam N., McGrath T., van der Pol C., Frank R., Prager R., Hare S., Dennie C. (2020). Thoracic Imaging Tests For The Diagnosis Of COVID-19. Cochrane Database Syst. Rev..

[39] World Health Organization (2020). Clinical Management of COVID-19: Interim Guidance, 27 May 2020.

[40] Bianco A., Buckley A.B., Overbey J., Smilen S., Wagner B., Dinglas C., Loudon H., Garely A., Brodman M., Stone J. (2020). Testing of Patients and Support Persons for Coronavirus Disease 2019 (COVID-19) Infection Before Scheduled Deliveries. Obstet. Gynecol..

[41] Knight M., Bunch K., Vousden N., Morris E., Simpson N., Gale C., O’Brien P., Quigley M., Brocklehurst P., Kurinczuk J.J. (2020). Characteristics and outcomes of pregnant women admitted to hospital with confirmed SARS-CoV-2 infection in UK: National population based cohort study. BMJ.

[42] Khoury R., Bernstein P.S., Debolt C., Stone J., Sutton D.M., Simpson L.L., Limaye M.A., Roman A.S., Fazzari M., Penfield C.A. (2020). Characteristics and Outcomes of 241 Births to Women With Severe Acute Respiratory Syndrome Coronavirus 2 (SARS-CoV-2) Infection at Five New York City Medical Centers. Obstet. Gynecol..

[43] Li J., Wang Y., Zeng Y., Song T., Pan X., Jia M., He F., Hou L., Li B., He S. (2020). Critically ill pregnant patient with COVID-19 and neonatal death within two hours of birth. Int. J. Gynaecol. Obstet..

[44] Alzamora M.C., Paredes T., Caceres D., Webb C.M., Valdez L.M., La Rosa M. (2020). Severe COVID-19 during pregnancy and possible vertical transmission. Am. J. Perinatol..

[45] Vallejo V., Ilagan J.G. (2020). A Postpartum Death Due to Coronavirus Disease 2019 (COVID-19) in the United States. Obstet. Gynecol..

[46] London V., McLaren R., Atallah F., Cepeda C., McCalla S., Fisher N., Stein J.L., Haberman S., Minkoff H. (2020). The Relationship between Status at Presentation and Outcomes among Pregnant Women with COVID-19. Am. J. Perinatol..

[47] Liu Y., Chen H., Tang K., Guo Y. (2020). Clinical manifestations and outcome of SARS-CoV-2 infection during pregnancy. J. Infect..

[48] Nepogodiev D., Bhangu A., Glasbey J.C., Li E., Omar O.M., Simoes J.F.F., Abbott T.E.F., Alser O., Arnaud A.P., Bankhead-Kendall B.K. (2020). Mortality and pulmonary complications in patients undergoing surgery with perioperative SARS-CoV-2 infection: An international cohort study. Lancet.

[49] Farghaly M., Kupferman F., Castillo F., Kim R. (2020). Characteristics Of Newborns Born To SARS-Cov-2-Positive Mothers: A Retrospective Cohort Study. Am. J. Perinat..

[50] Di Mascio D., Sen C., Saccone G., Galindo A., Grünebaum A., Yoshimatsu J., Stanojevic M., Kurjak A., Chervenak F., Suárez M.J.R. (2020). Risk Factors Associated With Adverse Fetal Outcomes In Pregnancies Affected By Coronavirus Disease 2019 (COVID-19): A Secondary Analysis Of The WAPM Study On COVID-19. JPM.

[51] Richtmann R., Torloni M.R., Oyamada Otani A.R., Levi J.E., Crema Tobara M., de Almeida Silva C., Dias L., Miglioli-Galvão L., Martins Silva P., Macoto Kondo M. (2020). Fetal deaths in pregnancies with SARS-CoV-2 infection in Brazil: A case series. Case Rep. Womens Health..

[52] Hachem R., Markou G., Veluppillai C., Poncelet C. (2020). Late miscarriage as a presenting manifestation of COVID-19. Eur. J. Obstet Gynecol. Reprod Biol..

[53] Yu N., Li W., Kang Q., Xiong Z., Wang S., Lin X., Liu Y., Xiao J., Liu H., Deng D. (2020). Clinical features and obstetric and neonatal outcomes of pregnant patients with COVID-19 in Wuhan, China: A retrospective, single-centre, descriptive study. Lancet Infect. Dis..

[54] Ferrazzi E., Frigerio L., Savasi V., Vergani P., Prefumo F., Barresi S., Bianchi S., Ciriello E., Facchinetti F., Gervasi M.T. (2020). Vaginal delivery in SARS-CoV-2-infected pregnant women in Northern Italy: A retrospective analysis. BJOG.

[55] Reis H.L.B.D., Boldrini N.A.T., Caldas J.V.J., Paz A.P.C.D., Ferrugini C.L.P., Miranda A.E. (2020). Severe coronavirus infection in pregnancy: Challenging cases report. Rev. Inst. Med. Trop Sao Paulo.

[56] Dong Y., Chi X., Hai H., Sun L., Zhang M., Xie W.F., Chen W. (2020). Antibodies in the breast milk of a maternal woman with COVID-19. Emer. Microbes Infect..

[57] Lowe B., Bopp B. (2020). COVID-19 vaginal delivery—A case report. Aust. N. Z. J. Obstet. Gynaecol..

[58] Bastug A., Hanifehnezhad A., Tayman C., Ozkul A., Ozbay O., Kazancioglu S., Bodur H. (2020). Virolactia In An Asymptomatic Mother With COVID-19. Breastfeed. Med..

[59] Hinojosa-Velasco A., de Oca P., García-Sosa L., Mendoza-Durán J., Pérez-Méndez M., Dávila-González E., Ramírez-Hernández D., García-Mena J., Zárate-Segura P., Reyes-Ruiz J. (2020). A Case Report Of Newborn Infant With Severe COVID-19 In Mexico: Detection Of SARS-Cov-2 In Human Breast Milk And Stool. IJID.

[60] World Health Organization Transmission of SARS-CoV-2: Implications for Infection Prevention Precautions. https://www.who.int/news-room/commentaries/detail/transmission-of-sars-cov-2-implications-for-infection-prevention-precautions.

[61] Khan S., Liu J., Xue M. (2020). Transmission of SARS-CoV-2, Required Developments in Research and Associated Public Health Concerns. Front. Med..

[62] Wiersinga W.J., Rhodes A., Cheng A.C., Peacock S.J., Prescott H.C. (2020). Pathophysiology, Transmission, Diagnosis, and Treatment of Coronavirus Disease 2019 (COVID-19): A Review. JAMA.

[63] Carducci A., Federigi I., Verani M. (2020). Covid-19 Airborne Transmission and Its Prevention: Waiting for Evidence or Applying the Precautionary Principle?. Atmosphere.

[64] Kotlyar A., Grechukhina O., Chen A., Popkhadze S., Grimshaw A., Tal O., Taylor H., Tal R. (2020). Vertical Transmission of Coronavirus Disease 2019: A Systematic Review And Meta-Analysis. Am. J. Obstet. Gynecol..

[65] Gajbhiye R., Modi D., Mahale S. (2020). Pregnancy outcomes, newborn complications and maternal-fetal transmission of SARS-CoV-2 in women withCOVID-19: A systematic review. medRxiv.

[66] Wang S., Guo L., Chen L., Liu W., Cao Y., Zhang J., Feng L. (2020). A Case Report of Neonatal 2019 Coronavirus Disease in China. Clin. Infect. Dis..

[67] Dhawan S., Pandey M. (2020). SARS-Cov-2 Vertical Transmission: Rare but A Potential Possibility. Indian J. Pediatr.

[68] Sisman J., Jaleel M., Moreno W., Rajaram V., Collins R., Savani R., Rakheja D., Evans A. (2020). Intrauterine transmission of SARS-CoV-2 infection in a preterm infant. Pediatr. Infect. Dis. J..

[69] Vivanti A.J., Vauloup-Fellous C., Prevot S., Zupan V., Suffee C., Do Cao J., Benachi A., De Luca D. (2020). Transplacental transmission of SARS-CoV-2 infection. Nat. Commun..

[70] Kulkarni R., Rajput U., Dawre R., Valvi C., Nagpal R., Magdum N., Vankar H., Sonkawade N., Das A., Vartak S. (2020). Early-Onset Symptomatic Neonatal COVID-19 Infection With High Probability Of Vertical Transmission. Infection.

[71] Patanè L., Morotti D., Giunta M.R., Sigismondi C., Piccoli M.G., Frigerio L., Mangili G., Arosio M., Cornolti G. (2020). Vertical transmission of COVID-19: SARS-CoV-2 RNA on the fetal side of the placenta in pregnancies with COVID-19 positive mothers and neonates at birth. Am. J. Obstet. Gynecol. MFM.

[72] Penfield C.A., Brubaker S.G., Limaye M.A., Lighter J., Ratner A.J., Thomas K.M., Meyer J., Roman A.S. (2020). Detection of SARS-COV-2 in Placental and Fetal Membrane Samples. Am. J. Obstet. Gynecol. MFM.

[73] Ferraiolo A., Barra F., Kratochwila C., Paudice M., Vellone V., Godano E., Varesano S., Noberasco G., Ferrero S., Arioni C. (2020). Report of Positive Placental Swabs For SARS-Cov-2 In An Asymptomatic Pregnant Woman With COVID-19. Medicina.

[74] Menter T., Mertz K., Jiang S., Chen H., Monod C., Tzankov A., Waldvogel S., Schulzke S., Hösli I., Bruder E. (2020). Placental Pathology Findings During and After SARS-Cov-2 Infection: Features Of Villitis And Malperfusion. Pathobiology.

[75] Zeng L., Xia S., Yuan W., Yan K., Xiao F., Shao J., Zhou W. (2020). Neonatal Early-Onset Infection With SARS-CoV-2 in 33 Neonates Born to Mothers With COVID-19 in Wuhan, China. JAMA Pediatr..

[76] Mohakud N., Yerru H., Rajguru M., Naik S. (2020). An Assumed Vertical Transmission of SARS-Cov-2During Pregnancy: A Case Report & Review of Literature. Cureus.

[77] Simões E., Silva A.C., Leal C.R.V. (2020). Is SARS-CoV-2 Vertically Transmitted?. Front. Pediatr..

[78] Dong L., Tian J., He S., Zhu C., Wang J., Liu C., Yang J. (2020). Possible Vertical Transmission of SARS-CoV-2 From an Infected Mother to Her Newborn. JAMA.

[79] Zeng H., Xu C., Fan J., Tang Y., Deng Q., Zhang W., Long X. (2020). Antibodies in Infants Born to Mothers With COVID-19 Pneumonia. JAMA.

[80] Fenizia C., Biasin M., Cetin I., Vergani P., Mileto D., Spinillo A., Gismondo M., Perotti F., Callegari C., Mancon A. (2020). Analysis of SARS-CoV-2 vertical transmission during pregnancy. Nat. Commun..

[81] Centers for Disease Control and Prevention Evaluation and Management Considerations for Neonates At Risk for COVID-19. https://www.cdc.gov/coronavirus/2019-ncov/hcp/caring-for-newborns.html.

[82] Wu Y.T., Liu J., Xu J.J., Chen Y.F., Yang W., Chen Y., Li C., Wang Y., Liu H., Zhang C. (2020). Neonatal outcome in 29 pregnant women with COVID-19: A retrospective study in Wuhan, China. PLoS Med..

[83] Hu X., Gao J., Luo X., Feng L., Liu W., Chen J., Benachi A., De Luca D., Chen L. (2020). Severe Acute Respiratory Syndrome Coronavirus 2 (SARS-Cov-2) Vertical Transmission In Neonates Born To Mothers With Coronavirus Disease 2019 (COVID-19) Pneumonia. Obstet. Gynecol..

[84] Marzollo R., Aversa S., Prefumo F., Saccani B., Perez C., Sartori E., Motta M. (2020). Possible Coronavirus Disease 2019 Pandemic And Pregnancy. Pediatr. Infect. Dis. J..

[85] Poon L.C., Yang H., Kapur A., Melamed N., Dao B., Divakar H., McIntyre H.D., Kihara A.B., Ayres-de-Campos D., Ferrazzi E.M. (2020). Global interim guidance on coronavirus disease 2019 (COVID-19) during pregnancy and puerperium from FIGO and allied partners: Information for healthcare professionals. Int. J. Gynaecol. Obstet..

[86] Qi H., Luo X., Zheng Y., Zhang H., Li J., Zou L., Feng L., Chen D., Shi Y., Tong C. (2020). Safe delivery for pregnancies affected by COVID-19. BJOG.

[87] Centers for Disease Control and Prevention Considerations for Inpatient Obstetric Healthcare Settings. https://www.cdc.gov/coronavirus/2019-ncov/hcp/inpatient-obstetric-healthcare-guidance.html.

[88] Royal College of Obstetricians & Gynaecologists Coronavirus (COVID-19) Infection in Pregnancy. https://www.rcog.org.uk/coronavirus-pregnancy.

[89] The Amercian College of Obstetricians & Gynaecologists Novel Coronavirus (COVID-19). https://www.acog.org/clinical/clinical-guidance/practice-advisory/articles/2020/03/novel-coronavirus-2019.

[90] Centers for Disease Control and Prevention Pregnancy, Breastfeeding, and Caring for Newborns. https://www.cdc.gov/coronavirus/2019-ncov/need-extra-precautions/pregnancy-breastfeeding.html.

[91] COVID-19 Treatment Guidelines Panel Coronavirus Disease 2019 (COVID-19) Treatment Guidelines. National Institutes of Health..

[92] The American College of Obstetricians and Gynecologists COVID-19 FAQs for Obstetrician-Gynecologists, Obstetrics. https://www.acog.org/clinical-information/physician-faqs/covid-19-faqs-for-ob-gyns-obstetrics.

[93] Saad A., Chappell L., Saade G., Pacheco L. (2020). Corticosteroids In The Management Of Pregnant Patients With Coronavirus Disease (COVID-19). Obstet. Gynecol..

[94] U.S. Food & Drug Administration FDA Approves First Treatment for COVID-19. https://www.fda.gov/news-events/press-announcements/fda-approves-first-treatment-covid-19.

[95] Pan H., Peto R., Karim Q., Alejandria M., Henao-Restrepo A., García C., Kieny M., Malekzadeh R., Murthy S., Preziosi M. (2020). Repurposed Antiviral Drugs For COVID-19 –Interim WHO SOLIDARITY Trial Results. medRxiv.

[96] Wu D., Fang D., Wang R., Deng D., Liao S. (2020). Management Of Pregnancy During The COVID-19 Pandemic. Glob. Chall..

[97] Mei Y., Luo D., Wei S., Liao X., Pan Y., Yang X., Lin Y. (2020). Obstetric Management Of COVID-19 In Pregnant Women. Front. Microbiol..

[98] Favilli A., Mattei Gentili M., Raspa F., Giardina I., Parazzini F., Vitagliano A., Borisova A.V., Gerli S. (2020). Effectiveness and safety of available treatments for COVID-19 during pregnancy: A critical review. J. Matern. Fetal. Neonatal. Med..

[99] Savasi V., Parisi F., Patanè L., Ferrazzi E., Frigerio L., Pellegrino A., Spinillo A., Tateo S., Ottoboni M., Veronese P. (2020). Clinical Findings And Disease Severity In Hospitalized Pregnant Women With Coronavirus Disease 2019 (COVID-19). Obstet. Gynecol..

[100] Naqvi M., Zakowski P., Glucksman L., Smithson S., Burwick R.M. (2020). Tocilizumab and Remdesivir in a Pregnant Patient With Coronavirus Disease 2019 (COVID-19). Obstet. Gynecol..

[101] Benhamou D., Keita H., Ducloy-Bouthors A., CARO Working Group (2020). Coagulation Changes And Thromboembolic Risk In COVID-19 Obstetric Patients. Anaesth. Crit. Care Pain Med..

[102] Hunt B., Retter A., McClintock C. (2020). Practical Guidance For The Prevention Of Thrombosis And Management Of Coagulopathy And Disseminated Intravascular Coagulation Of Patients Infected With COVID-19. JTH.

[103] D’Souza R., Malhamé I., Teshler L., Acharya G., Hunt B., McLintock C. (2020). A Critical Review Of The Pathophysiology Of Thrombotic Complications And Clinical Practice Recommendations For Thromboprophylaxis In Pregnant Patients With COVID-19. Acta Obstet. Gynecol. Scand..

[104] World Health Organization Breastfeeding and COVID-19. https://www.who.int/news-room/commentaries/detail/breastfeeding-and-covid-19.

[105] Centers for Disease Control and Prevention Coronavirus Disease (COVID-19) and Breastfeeding. https://www.cdc.gov/breastfeeding/breastfeeding-special-circumstances/maternal-or-infant-illnesses/covid-19-and-breastfeeding.html.

[106] UNICEF Breastfeeding during the COVID-19 pandemic. https://www.unicef.org/eap/breastfeeding-during-covid-19.

[107] Wang X., Zhou Z., Zhang J., Zhu F., Tang Y., Shen X. (2020). A Case of 2019 Novel Coronavirus in a Pregnant Woman With Preterm Delivery. Clin. Infect. Dis..

[108] Zhu H., Wang L., Fang C., Peng S., Zhang L., Chang G., Xia S., Zhou W. (2020). Clinical analysis of 10 neonates born to mothers with 2019-nCoV pneumonia. Transl. Pediatr..

[109] Wu Y., Liu C., Dong L., Zhang C., Chen Y., Liu J., Zhang C., Duan C., Zhang H., Mol B.W. (2020). Coronavirus disease 2019 among pregnant Chinese women: Case series data on the safety of vaginal birth and breastfeeding. BJOG..

[110] Yu Y., Li Y., Hu Y., Li B., Xu J. (2020). Breastfed 13 Month-Old Infant Of A Mother With COVID-19 Pneumonia: A Case Report. Int. Breastfeed. J..

[111] The American College of Obstetricians & Gynaecologists Coronavirus (COVID-19), Pregnancy, and Breastfeeding: A Message for Patients. https://www.acog.org/patient-resources/faqs/pregnancy/coronavirus-pregnancy-and-breastfeeding#How%20does%20COVID19%20affect%20pregnant%20women.

[112] Wu Y., Zhang C., Liu H., Duan C., Li C., Fan J., Li H., Chen L., Xu H., Li X. (2020). Perinatal depressive and anxiety symptoms of pregnant women during the coronavirus disease 2019 outbreak in China. Am. J. Obstet. Gynecol..

[113] Saccone G., Florio A., Aiello F., Venturella R., De Angelis M.C., Locci M., Bifulco G., Zullo F., Di Spiezio Sardo A. (2020). Psychological impact of coronavirus disease 2019 in pregnant women. Am. J. Obstet. Gynecol..

[114] Ng Q., Koh K., Tagore S., Mathur M. (2020). Perception and Feelings of Antenatal Women during COVID-19 Pandemic: A Cross-Sectional Survey. Ann. Acad Med. Singap..

[115] Lebel C., MacKinnon A., Bagshawe M., Tomfohr-Madsen L., Giesbrecht G. (2020). Elevated depression and anxiety among pregnant individuals during the COVID-19 pandemic. PsyArXiv.

[116] Zanardo V., Manghina V., Giliberti L., Vettore M., Severino L., Straface G. (2020). Psychological impact of COVID-19 quarantine measures in northeastern Italy on mothers in the immediate postpartum period. Int. J. Gynaecol. Obstet..

[117] Zhou Y., Shi H., Liu Z., Peng S., Wang R., Qi L., Li Z., Yang J., Ren Y., Song X. (2020). The Prevalence Of Psychiatric Symptoms Of Pregnant And Non-Pregnant Women During The COVID-19 Epidemic. Transl. Psychiatry.

[118] Effati-Daryani F., Zarei S., Mohammadi A., Hemmati E., Ghasemi Yngyknd S., Mirghafourvand M. (2020). Depression, Stress, Anxiety and Their Predictors In Iranian Pregnant Women During The Outbreak Of COVID-19. BMC Psychol..

[119] Christian L.M. (2015). Stress and Immune Function during Pregnancy: An Emerging Focus in Mind-Body Medicine. Curr Dir. Psychol Sci..

[120] Matvienko-Sikar K., Meedya S., Ravaldi C. (2020). Perinatal mental health during the COVID-19 pandemic. Women Birth..

[121] Burwick R., Yawetz S., Stephenson K., Collier A., Sen P., Blackburn B., Kojic E., Hirshberg A., Suarez J., Sobieszczyk M. (2020). Compassionate Use of Remdesivir in Pregnant Women With Severe Covid-19. Clin. Infect. Dis..

